# Incorporating Genomics and Bioinformatics across the Life Sciences Curriculum

**DOI:** 10.1371/journal.pbio.1000448

**Published:** 2010-08-10

**Authors:** Jayna L. Ditty, Christopher A. Kvaal, Brad Goodner, Sharyn K. Freyermuth, Cheryl Bailey, Robert A. Britton, Stuart G. Gordon, Sabine Heinhorst, Kelynne Reed, Zhaohui Xu, Erin R. Sanders-Lorenz, Seth Axen, Edwin Kim, Mitrick Johns, Kathleen Scott, Cheryl A. Kerfeld

**Affiliations:** 1 Department of Biology, University of St. Thomas, St. Paul, Minnesota, United States of America; 2 Department of Biological Sciences, St. Cloud State University, St. Cloud, Minnesota, United States of America; 3 Department of Biology, Hiram College, Hiram, Ohio, United States of America; 4 Biochemistry Department, University of Missouri-Columbia, Columbia, Missouri, United States of America; 5 Department of Biochemistry, University of Nebraska-Lincoln, Lincoln, Nebraska, United States of America; 6 Department of Microbiology and Molecular Genetics, Michigan State University, East Lansing, Michigan, United States of America; 7 Department of Biology, Presbyterian College, Clinton, South Carolina, United States of America; 8 Department of Chemistry and Biochemistry, The University of Southern Mississippi, Hattiesburg, Mississippi, United States of America; 9 Biology Department, Austin College, Sherman, Texas, United States of America; 10 Department of Biological Sciences, Bowling Green State University, Bowling Green, Ohio, United States of America; 11 Department of Microbiology, Immunology and Molecular Genetics, University of California – Los Angeles, Los Angeles, California, United States of America; 12 Department of Energy-Joint Genome Institute, Walnut Creek, California, United States of America; 13 Department of Biological Sciences, Northern Illinois University, DeKalb, Illinois, United States of America; 14 Department of Integrative Biology, University of South Florida, Tampa, Florida, United States of America; 15 Department of Plant and Microbial Biology, University of California Berkley, Berkeley, California, United States of America

## Introduction

Undergraduate life sciences education needs an overhaul, as clearly described in the National Research Council of the National Academies' publication *BIO 2010: Transforming Undergraduate Education for Future Research Biologists*. Among BIO 2010's top recommendations is the need to involve students in working with real data and tools that reflect the nature of life sciences research in the 21st century [1]. Education research studies support the importance of utilizing primary literature, designing and implementing experiments, and analyzing results in the context of a bona fide scientific question [1]–[12] in cultivating the analytical skills necessary to become a scientist. Incorporating these basic scientific methodologies in undergraduate education leads to increased undergraduate and post-graduate retention in the sciences [13]–[16]. Toward this end, many undergraduate teaching organizations offer training and suggestions for faculty to update and improve their teaching approaches to help students learn as scientists, through design and discovery (e.g., Council of Undergraduate Research [www.cur.org] and Project Kaleidoscope [ www.pkal.org]).

With the advent of genome sequencing and bioinformatics, many scientists now formulate biological questions and interpret research results in the context of genomic information. Just as the use of bioinformatic tools and databases changed the way scientists investigate problems, it must change how scientists teach to create new opportunities for students to gain experiences reflecting the influence of genomics, proteomics, and bioinformatics on modern life sciences research [17]–[41].

**Figure 1:**
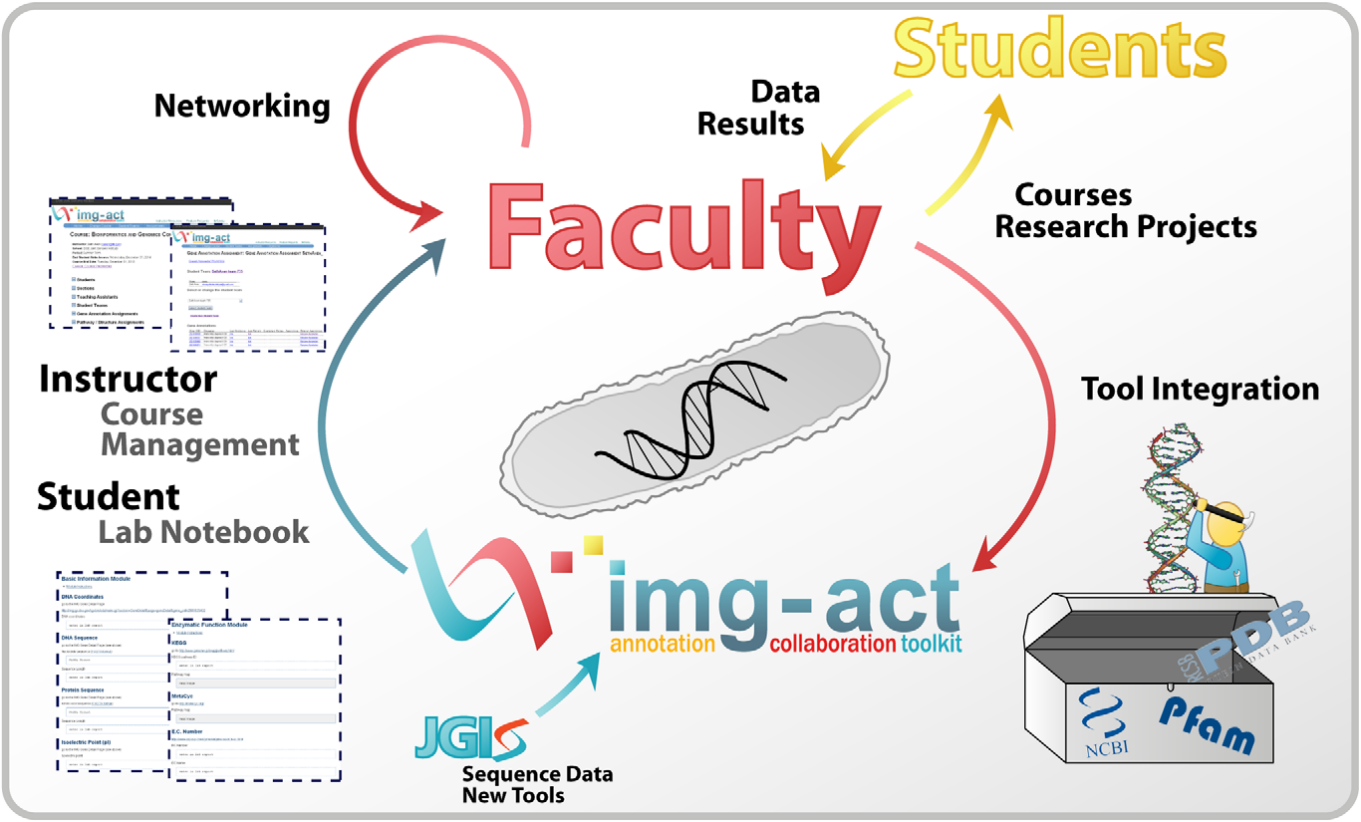
Overview of the IMG-ACT program. IMG-ACT was developed by instructors from diverse institutions in collaboration with the DOE-JGI. The program's purpose is to serve as a new bioinformatics platform to (1) provide faculty with sequence data and new bioinformatic tools, (2) develop on-line pedagogical tools for student data and course management, and (3) help innovate and update undergraduate education by serving as a clearinghouse for faculty networking and instruction for those new to the area of bioinformatics. Visit http://img-act.jgi-psf.org/tour for a tour of the IMG-ACT program, a sample annotation, and bioinformatic tutorials. To try gene annotation using the IMG-ACT tools, download a template notebook page at http://img-act.jgi-psf.org/tour/blank_notebook.rtf.

Educators have responded by incorporating bioinformatics into diverse life science curricula [42]–[44]. While these published exercises in, and guidelines for, bioinformatics curricula are helpful and inspirational, faculty new to the area of bioinformatics inevitably need training in the theoretical underpinnings of the algorithms [45]. Moreover, effectively integrating bioinformatics into courses or independent research projects requires infrastructure for organizing and assessing student work. Here, we present a new platform for faculty to keep current with the rapidly changing field of bioinformatics, the Integrated Microbial Genomes Annotation Collaboration Toolkit (IMG-ACT) (Figure 1). It was developed by instructors from both research-intensive and predominately undergraduate institutions in collaboration with the Department of Energy-Joint Genome Institute (DOE-JGI) as a means to innovate and update undergraduate education and faculty development. The IMG-ACT program provides a cadre of tools, including access to a clearinghouse of genome sequences, bioinformatics databases, data storage, instructor course management, and student notebooks for organizing the results of their bioinformatic investigations. In the process, IMG-ACT makes it feasible to provide undergraduate research opportunities to a greater number and diversity of students, in contrast to the traditional mentor-to-student apprenticeship model for undergraduate research, which can be too expensive and time-consuming to provide for every undergraduate.

The IMG-ACT serves as the hub for the network of faculty and students that use the system for microbial genome analysis. Open access of the IMG-ACT infrastructure to participating schools ensures that all types of higher education institutions can utilize it. With the infrastructure in place, faculty can focus their efforts on the pedagogy of bioinformatics, involvement of students in research, and use of this tool for their own research agenda. What the original faculty members of the IMG-ACT development team present here is an overview of how the IMG-ACT program has affected our development in terms of teaching and research with the hopes that it will inspire more faculty to get involved.

## Faculty Involved in the Project

The founding faculty members became involved in IMG-ACT for many different reasons. For some, this program was a natural extension of current work in genomics education and research [46]. These participants were drawn to a new platform with anticipated improvement in teaching and student assessment. Some without experience in the field got involved to bring new and updated course content to their curricula, either self-instigated or as mandated by departmental curricular reform. Regardless of the impetus, the common goal for everyone involved was to participate in a unique faculty development opportunity to incorporate novel research into undergraduate coursework. Appointments ranged from a post-doctoral associate, to lecturers, to tenure-track faculty from either predominantly undergraduate or research-intensive institutions. Faculty came from a wide variety of life-science departments with teaching responsibilities in various disciplines including standard undergraduate courses in molecular biology, biochemistry, cell biology, genetics, and microbiology to more applied courses in bioinformatics, genomics and genome annotation, and independent study and research experiences.

## Teaching Enhancement

Designed to be flexible, the IMG-ACT platform can be used to illustrate a few key concepts in an introductory course, serve as the foundation for an entire course in bioinformatics or a microbial genome annotation research project. The main goal has been to engage students in the scientific method using real data, which exposes them to the ambiguity inherent in discovering and organizing new information. For example, in introductory science courses, instructors have utilized IMG-ACT as an active learning tool for the annotation of genes to master basic molecular concepts of gene and operon structure. In upper division courses, the IMG-ACT program has been used as a foundation for larger projects including annotating pathways or the selection of an entire genome for annotation. Such experiences drove the development of multiple new undergraduate courses at predominantly undergraduate institutions (Text S1).

Key benefits to faculty that utilize IMG-ACT are the bioinformatic networking and educational resources that are available. The Web-distributed nature of the IMG-ACT platform facilitates the exchange of resources, ideas, and experiences of talented and committed educators as well as stimulating collaboration across multiple institutions. Tutorial resources are available for anyone interested in using IMG-ACT (http://img-act.jgi-psf.org/tour/modules), reducing the time needed for new course development.

Due to the interdisciplinary nature of bioinformatics, IMG-ACT can be used to promote connections between science subject areas. For example, Austin College established teamwork between undergraduate microbiology and chemistry courses on a project where the microbiology group annotated carotenoid biosynthetic pathways in *Planctomyces limnophilus* and the chemistry group purified the pigment from the reddish-colored colonies. Interestingly, although the students were able to identify genes for lycopene biosynthesis in *P. limnophilus*, experiments conducted in the chemistry course showed that the pigment produced by the organism did not appear to be lycopene but may be some other, as of yet unidentified, pigment. This is one example of how hypotheses developed from genome studies in one course might be extended to functional studies within not only the same courses, but also in other disciplines as well.

To reflect the learner-centered nature of using gene annotation to teach undergraduates, the use of IMG-ACT has resulted in the incorporation of new teaching pedagogy at various institutions. At UCLA, an interdepartmental laboratory curriculum is under development in which all life science majors participate in a research experience, and annotation using the IMG-ACT platform was chosen as one means to reach this goal. To manage the large group of students engaging in annotation, UCLA is using peer-mentoring instruction [47]–[50], in which two or three students meet with instructors for annotation tutorial instruction and develop tutorials (which are available on the UCLA website at http://www.mimg.ucla.edu/faculty/sanderslorenz/education.html) to present to their classmates. This approach provides each lab section with at least one student “expert” on the annotation tools and concepts and builds the idea of team learning: students mentor one another and build a community of local peer experts in bioinformatics, modeling that science is a collaborative effort.

## Research Development

Faculty participation in the IMG-ACT program has also been instrumental in the enhancement of faculty research (see Text S1). Involvement with IMG-ACT has either helped support current research programs or has opened new avenues of research for some involved with the project. In addition, the use of IMG-ACT for undergraduate research can be used to strengthen the broader impacts of research agendas. Student annotations have generated preliminary data for research grant proposals, and the educational and outreach strengths of the IMG-ACT program contribute to the promotion of teaching, training, and learning and enhance the infrastructure of research and education in building networks and partnerships between universities. The National Science Foundation recently funded a collaborative research grant between the University of St. Thomas and the University of California, Davis (Proposal 0919930) whereby St. Thomas undergraduates are responsible for the annotation of the *P. putida* F1 genome and of target genes identified in toluene-induced microarray analysis. In addition, these students will be responsible in part for the functional genomics research projects that evolve from these various annotations. Due to the sheer volume of genomes that are available for annotation, the breadth of genomes in terms of representative domains and the diversity of lifestyles that the DOE-JGI Genomic Encyclopedia of Bacteria and Archaea (GEBA) ([51],[52], see also www.jgi.doe.gov/programs/GEBA/pilot.html) project offers, the number of new research possibilities is virtually endless.

## Conclusion

We have described a new paradigm for faculty development and undergraduate education in Bioinformatics. IMG-ACT is a response to the need to update undergraduate curriculum with genomics and bioinformatics by combining genome analysis with instruction. This collaborative platform meets established goals in pedagogy of the scientific method while providing an authentic research experience. IMG-ACT provides affordable instructional resources and a plethora of future uses for educators/researchers in all academic arenas. The system is continually evolving in response to the needs of its registered users; they are able to make feature requests for the system that are implemented in frequent updates (averaging six releases per year). Involvement in, and interaction with, the IMG-ACT program has also produced unanticipated benefits for the faculty (see Text S1). Since its development in 2008, the IMG-ACT system has been used by nearly 100 faculty members and over 1,600 students nationwide. To explore the IMG-ACT system and annotate an IMG gene of your choice, visit http://img-act.jgi-psf.org/user/login. To apply to participate in the faculty training, visit http://www.jgi.doe.gov/education/genomeannotation.html.

## Supporting Information

Text S1 **Course development, grant proposals, and other corollary benefits enabled by the program.**
(0.03 MB DOC)

## Abbreviations

IMG-ACT: Integrated Microbial Genomes Annotation Collaboration Toolkit
