# Supplementary material for: Incorporating Genomics and Bioinformatics across the Life Sciences Curriculum
Source: PLoS Biol. 2010 Aug 10;8(8):e1000448. doi: 10.1371/journal.pbio.1000448 (PMC2919421; doi:10.1371/journal.pbio.1000448)
Supplement: Text S1 — Course development, grant proposals, and other corollary benefits enabled by the program. (0.03 MB DOC) [file pbio.1000448.s001.doc]

**Supporting Information**

**Course Development**

New introductory and advanced courses in Bioinformatics, Genomics, and Interdisciplinary STEM courses were developed at the following institutions using IMG-ACT: Austin College, Bowling Green State University, Michigan State University, Univ. of California, Los Angeles, Univ. Nebraska-Lincoln, Univ. South Florida, Univ. of St. Thomas, and Presbyterian College.

**Educational Grants (*pending)**

IMG-ACT supported and was an integral part of the following educational grant initiatives: a Li-Cor instrumentation grant at Austin College and the Univ. of St. Thomas; a collaborative DOE-META grant to fund a parallel platform for metagenomics at Hiram College, St. Cloud State Univ., and the DOE-JGI; internal funding for a genomics wet lab at Michigan State Univ.; an NSF RCN-UBE Microbial Genome Annotation Network grant at St. Cloud State Univ.; a HHMI Undergraduate Science Education Grant* and NSF CCLI* grants at Univ. of California, Los Angeles; and a USDA Higher Education Challenge Grant at the Univ. of Missouri-Columbia.

**Research Grants (*pending)**

IMG-ACT supported and was an integral part of the broader impacts for the following research grant initiatives: a collaborative grant proposal* on the metagenomic analysis of microbial communities on maize leaves at Hiram College, Univ. of North Carolina-Wilmington and Presbyterian College and a collaborative NSF grant to investigate aromatic hydrocarbon chemotaxis at the Univ. of St. Thomas and Univ. of California-Davis.

**Corollary Benefits**

Increased visibility of schools and programs within and outside of respective universities has occurred through press releases in local and internal newspapers at Bowling Green Univ. ("Biology Students to Take Part in Genome Project" <http://www.bgsu.edu/offices/mc/news/2007/news42590.html>) and Univ. of California, Los Angeles ("UCLA Science Students in Pilot Federal Program Will Conduct Original Research in Laboratory Classes"  <http://www.newsroom.ucla.edu/portal/ucla/UCLA-Science-Students-in-Pilot-8231.aspx> and "College Students Engage in Research in Pilot Program" <http://www.nsta.org/publications/news/story.aspx?id=54427>), invitations to speak about the program, and exposure of IMG-ACT through student presentations at local and regional meetings at Univ. Nebraska-Lincoln, Bowling Green Univ. (Fig. 2), and the Univ. of St. Thomas.
